# LIPI及iSEND免疫评分在晚期非小细胞肺癌免疫治疗中的预测价值分析

**DOI:** 10.3779/j.issn.1009-3419.2022.102.47

**Published:** 2022-11-20

**Authors:** 怡颖 罗, 新娟 王, 一博 王, 国俊 张

**Affiliations:** 450000 郑州，郑州大学第一附属医院呼吸与危重症科 Department of Pulmonary and Critical Care Medicine, The First Affiliated Hospital of Zhengzhou University, Zhengzhou 450000, China

**Keywords:** 肺肿瘤, 肺免疫预后指数, iSEND免疫评分, 免疫检查点抑制剂, 预后, Lung neoplasms, Lung immune prognostic index, iSEND immune score, Immune checkpoint inhibitors, Prognosis

## Abstract

**背景与目的:**

本研究回顾性分析了肺癌免疫治疗预后指数（lung immune prognostic index, LIPI）及iSEND免疫评分系统在中国晚期非小细胞肺癌（non-small cell lung cancer, NSCLC）患者免疫治疗中的应用价值，以寻找指导临床制定NSCLC治疗方案的新指标。

**方法:**

回顾性分析178例应用免疫治疗的晚期NSCLC患者的临床资料。对患者分别进行LIPI及iSEND免疫评分，绘制受试者工作特征曲线（receiver operating characteristic curve, ROC）并比较两者对客观缓解率（objective response rate, ORR）、疾病控制率（disease control rate, DCR）、无进展生存期（progression free survival, PFS）的预测价值。应用*Kaplan-Meier*方法进行生存分析，单因素和多因素*Cox*回归进行各种临床特征与PFS之间的关系分析。

**结果:**

iSEND免疫评分预测ORR、DCR、PFS的曲线下面积（area under the cuever, AUC）分别为0.616、0.634、0.631；LIPI预测的AUC分别为0.789、0.750、0.732，均大于iSEND免疫评分（*P* < 0.05）。LIPI评分良好、中等、较差组患者的中位PFS分别为9.9个月、6.1个月、3.7个月；iSEND免疫评分良好、中等、较差组患者中位PFS分别为9.9个月、7.0个月、3.5个月，差异均具有统计学意义（*P* < 0.001）。在免疫联合治疗亚组中不同LIPI及不同iSEND免疫评分分组患者中位PFS差异同样具有统计学意义（*P* < 0.05）。*Cox*回归分析示衍生中性粒细胞与淋巴细胞的比率（derived neutrophil-to-lymphocyte ratio, dNLR）、乳酸脱氢酶（lactic dehydrogenase, LDH）与PFS独立相关（*P* < 0.05）。

**结论:**

LIPI及iSEND免疫评分系统均能有效预测晚期NSCLC免疫治疗疗效及预后，且LIPI较iSEND免疫评分具有更高的预测价值。

## Abstract (en)

**Background and objective:**

This study retrospectively analyzed the application value of lung cancer immunotherapy prognostic index (LIPI) and iSEND immune scoring system in advanced non-small cell lung cancer (NSCLC) patients treated with immunotherapy in China, in order to find guidance for clinical development of NSCLC treatment plan.

**Methods:**

The clinical data of 178 patients with advanced NSCLC treated with immunotherapy were analyzed retrospectively. LIPI and iSEND immune scores were performed, receiver operating characteristic (ROC) curves were drawn, and the predictive values of two models for objective response rate (ORR), disease control rate (DCR), and progression-free survival (PFS) were compared. *Kaplan-Meier* method was used for survival analysis, and *Cox* regression analysis method was used for univariate analysis and multivariate analysis.

**Results:**

The area under the curver (AUC) of ORR, DCR and PFS predicted by iSEND immune score were 0.616, 0.634 and 0.631 respectively; LIPI were 0.789, 0.750 and 0.732 respectively, which were higher than iSEND immune score (*P* < 0.05). The median PFS of patients in LIPI score groups 0, 1 and 2 were 9.9 months, 6.1 mon and 3.7 mon respectively; The median PFS of patients with good, moderate and poor iSEND immune scores were 9.9 mon, 7.0 mon and 3.5 mon respectively, with statistically significant differences (*P* < 0.001). In the immunotherapy subgroup, the median PFS of patients with different LIPI and iSEND immune scores was also statistically significant. *Cox* regression analysis showed that the derived neutrophil to lymphocyte ratio (dNLR), lactic dehydrogenase (LDH) and PFS were independently correlated (*P* < 0.05).

**Conclusion:**

LIPI and iSEND immune scoring system can effectively predict the efficacy and prognosis of advanced NSCLC treated with immunotherapy, and LIPI has higher predictive value than iSEND immune scoring system.

肺癌是癌症相关死亡的主要原因之一^[1]^，病理类型为非小细胞肺癌（non-small cell lung cancer, NSCLC）的患者占其主要部分（80%-85%）^[2]^。免疫检查点抑制剂（immune checkpoint inhibitors, ICIs）等的出现将肺癌治疗带入免疫治疗时代。虽然NSCLC患者应用免疫治疗组较化疗组有明显的受益，但仍有部分患者无法从中获益。目前仍缺乏免疫治疗疗效特异性标志物，难以从临床中筛选出免疫治疗优势人群。肺癌免疫治疗预后指数（lung immune prognostic index, LIPI）由Mezquita等^[3]^提出，并发现LIPI评分与NSCLC应用免疫治疗预后相关。Park等^[4]^为快速、有效地筛选出ICIs耐药人群，开发出iSEND免疫评分模型。两种模型均用于NSCLC免疫治疗疗效预测，但尚无研究进行两种模型预测能力的比较，且两种模型对中国晚期NSCLC患者应用免疫治疗疗效及预后的预测价值仍需进一步验证。

LIPI^[3]^由治疗前衍生中性粒细胞与淋巴细胞的比率（derived neutrophil-to-lymphocyte ratio, dNLR）及乳酸脱氢酶（lactic dehydrogenase, LDH）组成。iSEND免疫评分^[4]^是由患者性别、美国东部肿瘤协作组体力状况评分（Eastern Cooperative Oncology Group performance status, ECOG PS）、基线中性粒细胞/淋巴细胞比值（neutrophil-to-lymphocyte ratio, NLR）、DNLR（第2周期免疫治疗前NLR和基线NLR之差）构成。本研究旨在分析并对比LIPI及iSEND免疫评分对中国晚期NSCLC患者免疫治疗疗效及预后的预测价值。

## 1 资料与方法

### 1.1 研究对象

回顾性收集于2019年1月1日-2021年7月1日于我院行ICIs治疗的NSCLC患者的临床资料。纳入标准：①18岁以上的成人患者；②组织病理确诊为IIIB期-IV期NSCLC；③接受ICIs治疗大于2个周期；④无严重的重要脏器合并症。排除标准：①双原发肿瘤；②疗效评价信息缺失；③免疫性疾病及血液病病史；④对免疫治疗过敏或不耐受；⑤表皮生长因子受体（epidermal growth factor receptor, *EGFR*）、间变性淋巴瘤激酶（anaplastic lymphoma kinase, *ALK*）、c-ros肉瘤致癌因子-受体酪氨酸激酶（ROS proto-oncogene 1 receptor tyrosine kinase, *ROS1*）等驱动基因阳性。最终共纳入178例。本研究得到郑州大学第一附属医院伦理委员会批准（No.2021-KY-0302-002）。

### 1.2 资料收集

从电子病例系统收集患者的一般临床资料，包括性别、年龄、吸烟史、免疫治疗线数、免疫治疗方式、ECOG PS等。检查检验信息包括行免疫治疗前1周内最近1次血常规及第2周期治疗前血常规、免疫治疗前LDH、肿瘤病理类型、影像学检查等。根据血常规结果计算其他血液炎性标志物水平并进行LIPI及iSEND免疫评分。LIPI：①dNLR > 3=1分、dNLR≤3=0分；②LDH > 245 IU/L=1分，LDH≤245 IU/L=0分；总分=0为良好组，总分=1为中等组，总分=2为不良组。iSEND免疫评分：①男性=1分，女性=0分；②ECOG PS≥2=1分，ECOG PS < 2=0分；③NLR≥5且DNLR≥0=2分，其他=0分；总分=0为良好组，总分=1为中等组，总分≥2为不良组。

### 1.3 疗效评估及随访

通过电子病历或电话咨询方式进行随访，末次随访时间为2022年6月15日。通过患者的影像学检查[胸部计算机断层扫描（computed tomography, CT）、腹部CT、头颅磁共振成像（magnetic resonance imaging, MRI）、骨扫描、正电子发射计算机断层显像（positron emission tomography-CT, PET-CT）等]进行疗效评价。疗效评价采用实体瘤疗效评估标准1.1版，分为完全缓解（complete response, CR）、部分缓解（partial response, PR）、疾病稳定（stable disease, SD）和疾病进展（progressive disease, PD）。客观缓解率（objective response rate, ORR）=（CR+PR）例数/总例数×100%。疾病控制率（disease control rate, DCR）=（CR+PR+SD）例数/总例数×100%。无进展生存期（progression-free survival, PFS）定义为接受免疫治疗开始到观察到疾病进展或者因任何原因死亡的时间。本研究以ORR及DCR评价疗效，以PFS评价预后。

### 1.4 统计学分析

应用SPSS 24.0软件进行分析与绘图，Medcalc 19.20软件比较曲线下面积（area under the curve, AUC）的差异。绘制受试者工作特征曲线（receiver operating characteristic curve, ROC），以AUC评价预测效能。应用*Kaplan-Meier*法进行生存分析，*Log-rank*检验进行组间比较。*Cox*比例风险模型进行单因素及多因素分析。*P* < 0.05为差异有统计学意义，*P*值均为双侧检验。

## 2 结果

### 2.1 患者一般临床特征

共有178例患者纳入本研究。65岁以下患者占多数为115例（64.6%）；男性较多为144例（80.9%）；有75例患者具有吸烟史（42.1%）；ECOG PS评分为0分-1分的患者占多数，共147例（82.6%）。IV期患者109例（61.2%）。鳞癌74例（41.6%），非鳞癌104例（58.4%）；56例（31.5%）患者免疫治疗为一线治疗；仅1例应用程序性死亡配体1（programmed cell death ligand 1, PD-L1）单抗治疗，其余177例均应用程序性死亡受体1（programmed cell death 1, PD-1）单抗治疗；免疫单药治疗26例（14.6%），联合治疗152例（85.4%）；联合治疗的患者中ICIs+化疗85例（47.8%），ICIs+抗血管药物48例（27.0%），ICIs+化疗+抗血管药物19例（10.7%）；109例（61.2%）患者DNLR < 0，69例（38.8%）≥0；130例（73.0%）患者NLR < 5；104例（58.4%）例患者dNLR > 3；65例（36.5%）患者LDH > 245 IU/L；应用免疫治疗后，无CR病例，72例（40.4%）患者达到PR，55例（30.9%）患者达到SD，PD患者51例（28.7%）（表 1）。

**表 1:** 178例患者的临床特征 Clinical characteristics of 178 patients

Characteristics		*n*	%
Age	< 65 yr	115	64.6
	≥65 yr	63	35.4
Gender	Female	34	19.1
	Male	144	80.9
ECOG PS	0-1	147	82.6
	2	31	17.4
Smoking	No	103	57.9
	Yes	75	42.1
Histology	Squamous carcinoma	74	41.6
	Non-squamous carcinoma	104	58.4
Lines of treatment	1	56	31.5
	≥2	122	68.5
TNM stage	III	69	38.8
	IV	109	61.2
Option of treatment	Monotherapy	26	14.6
	Combination therapy	152	85.4
Best overall response	PR	72	40.4
	SD	55	30.9
	PD	51	28.7
DNLR	< 0	109	61.2
	≥0	69	38.8
NLR	< 5	130	73.0
	≥5	48	27.0
dNLR	≤3	74	41.6
	> 3	104	58.4
LDH	≤245 IU/L	113	63.5
	> 245 IU/L	65	36.5
ECOG PS: Eastern Cooperative Oncology Group performance status; TNM: tumor-node-metastasis; NLR: neutrophil-to-lymphocyte ratio; dNLR: derived neutrophil-to-lymphocyte ratio; LDH: lactic dehydrogenase; PR: partial response; SD: stable disease; PD: progressive disease.			

### 2.2 ROC曲线分析

ROC曲线分析显示，iSEND免疫评分预测ORR、DCR、PFS的AUC分别为0.616、0.634、0.631，LIPI评分预测ORR、DCR、PFS的AUC分别为0.789、0.750、0.732，均大于iSEND免疫评分（*P* < 0.05）（表 2，图 1）。

**表 2:** LIPI及iSEND免疫评分对ORR、DCR、PFS的预测价值 The predictive value of LIPI and iSEND immune scores on ORR, DCR and PFS

Index	Predictive models	AUC	95%CI	*P*
ORR	LIPI	0.789	0.720-0.857	< 0.001
	iSEND	0.616	0.534-0.698	
DCR	LIPI	0.750	0.671-0.830	0.015
	iSEND	0.634	0.539-0.729	
PFS	LIPI	0.732	0.658-0.805	0.017
	iSEND	0.631	0.549-0.713	
LIPI: lung immune prognostic index; ORR: objective response rate; DCR: disease control rate; PFS: progression-free survival.				

**图 1:**
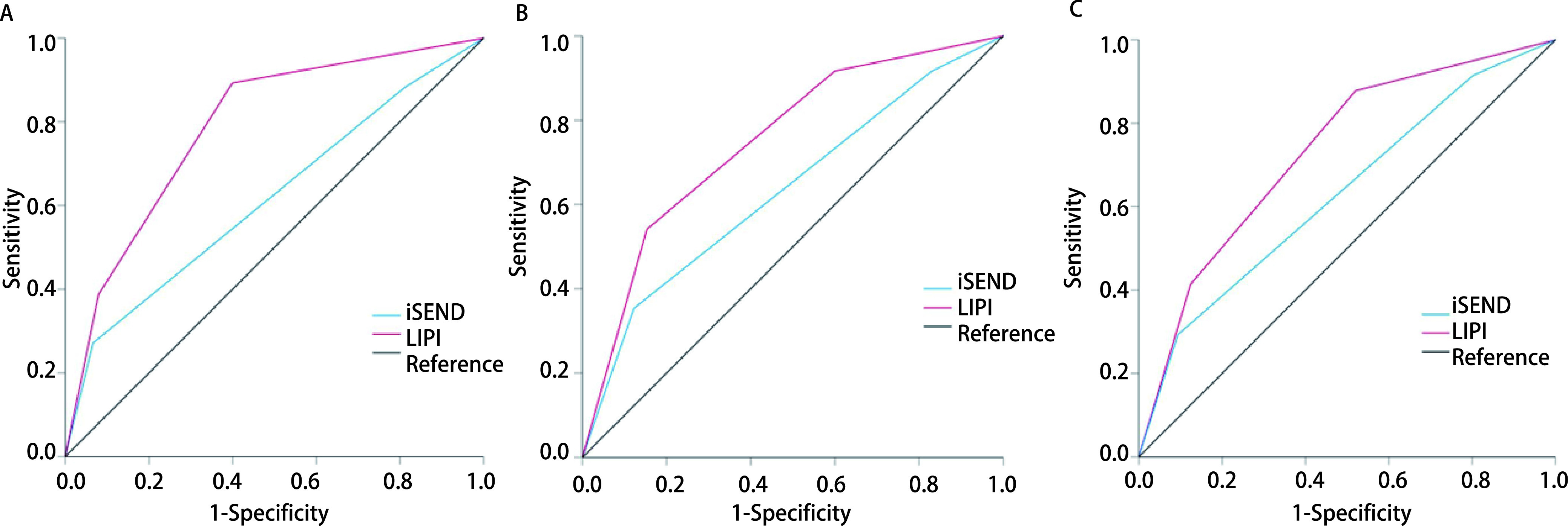
受试者工作特征曲线分析。A：LIPI、iSEND免疫评分系统对ORR预测的ROC曲线。B：LIPI、iSEND免疫评分系统对DCR预测的ROC曲线。C：LIPI、iSEND免疫评分系统对6个月时PFS预测的ROC曲线。 Receiver operating characteristic (ROC) analysis. A: ROC curve predicted by LIPI and iSEND immune scoring system to ORR. B: ROC curve predicted by LIPI and iSEND immune scoring system to DCR. C: ROC curve predicted by LIPI and iSEND immune scoring system for PFS at 6 mon.

### 2.3 生存分析

全组178例NSCLC患者的中位随访时间为19.23（16.65-21.81）个月，中位PFS为6.4（5.4-7.5）个月。生存分析示：①LIPI不良组PFS为3.7个月，中等组为6.1个月，良好组为9.9个月，差异具有统计学意义（*P* < 0.05）（表 3，图 2A）；②iSEND免疫评分不良组PFS为3.5个月，中等组为7.0个月，良好组为9.9个月，差异具有统计学意义（*P* < 0.05）（表 3，图 2B）。在26例免疫单药治疗的患者中，不同iSEND免疫评分组及不同LIPI组的PFS无明显差异（*P* > 0.05）。在152例免疫联合治疗患者中，生存分析示：①LIPI不良组PFS为4.0个月，中等组为5.9个月，良好组为10.7个月，差异具有统计学意义（*P* < 0.05）（表 4，图 2C）。②iSEND免疫评分不良组PFS为3.4个月，中等组为7.4个月，良好组为11.0个月，差异具有统计学意义（*P* < 0.05）（表 4，图 2D）。应用*Cox*回归分析分别在全组患者及免疫联合治疗亚组中对PFS进行单因素和多因素分析，纳入因素包括年龄、性别、ECOG PS评分、吸烟史、病理类型、肿瘤分期、是否一线治疗、治疗方式、DNLR、NLR、dNLR、LDH。免疫联合治疗亚组回归分析结果与全组患者一致，单因素分析中发现ECOG PS评分0分-1分、NLR < 5、dNLR≤3、LDH≤245 IU/L与较长的PFS相关（*P* < 0.05）；多因素分析结果表明，dNLR≤3、LDH≤245与较长的PFS独立相关（*P* < 0.05）（表 5，表 6）。

**表 3:** 178例免疫治疗NSCLC患者LIPI、iSEND免疫评分与mPFS的关系 Relationship between LIPI, iSEND immune score and mPFS in 178 treated with immunotherapy NSCLC patients

Predictive models	Group	*n*	mPFS (mon)	95%CI
LIPI	Good	56	9.9	8.453-11.247
	Intermediate	76	6.1	4.408-7.792
	Poor	46	3.7	2.738-4.687
iSEND	Good	26	9.9	7.760-11.940
	Intermediate	119	7.0	5.716-8.280
	Poor	33	3.5	2.583-4.382
Total		178	6.4	5.425-7.454
NSCLC: non-small cell lung cancer; mPFS: median PFS.				

**图 2:**
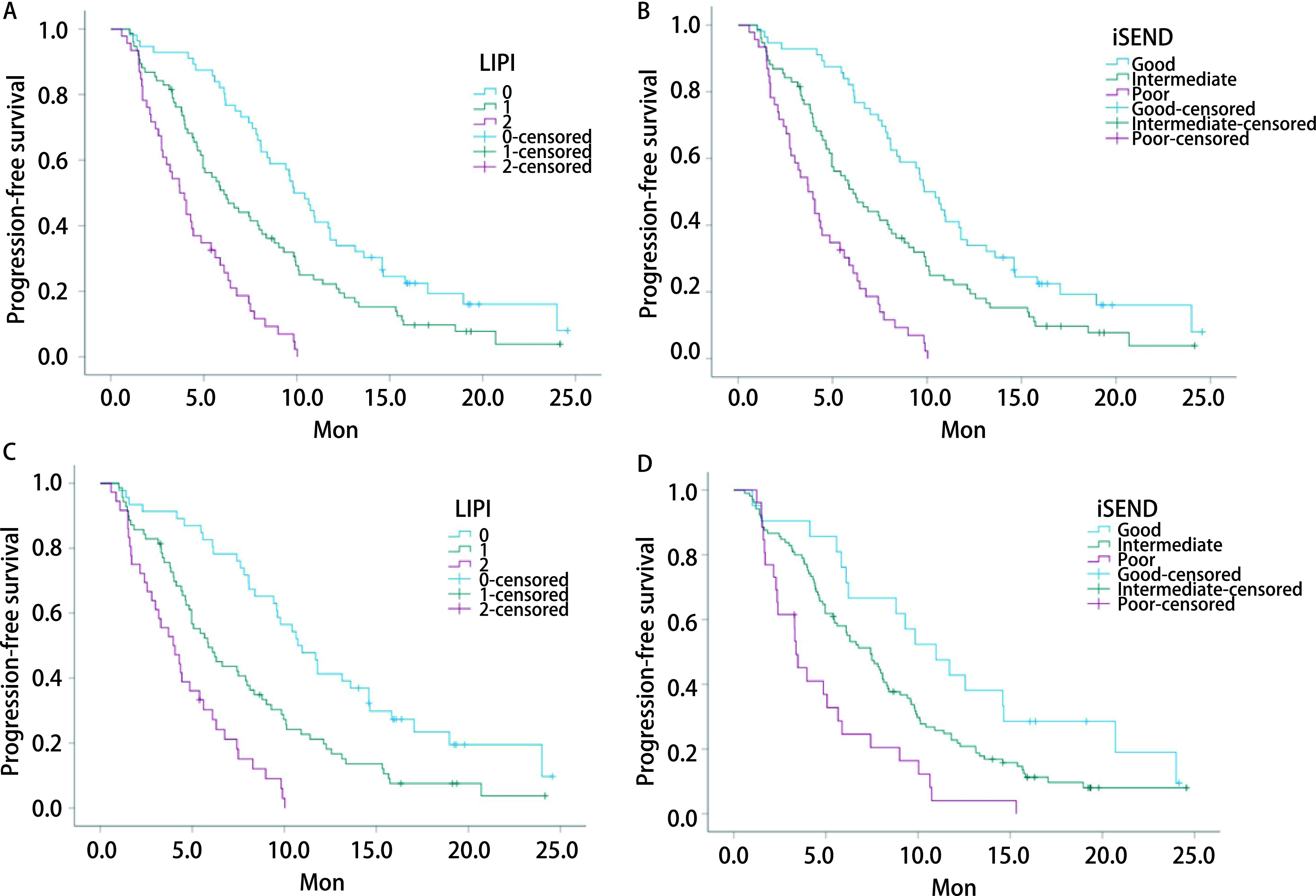
PFS生存曲线。A：不同LIPI评分分组免疫治疗患者的生存曲线。B：不同iSEND免疫评分分组免疫治疗患者的生存曲线。C：不同LIPI评分分组免疫联合治疗患者的生存曲线。D：不同iSEND免疫评分分组免疫联合治疗患者的生存曲线。 *Kaplan-Meier* survival analysis for PFS. A: *Kaplan-Meier* curves for PFS in patients treated with immunotherapy grouped by LIPI. B: *Kaplan-Meier* curves for PFS in patients treated with immunotherapy grouped by iSEND immune score. C: *Kaplan-Meier* curves for PFS in patients treated with combined immunotherapy patients grouped by LIPI. D: *Kaplan-Meier* curves for PFS in patients treated with combined immunotherapy grouped by iSEND immune score.

**表 4:** 152例免疫联合治疗NSCLC患者LIPI、iSEND免疫评分与PFS的关系 Relationship between LIPI, iSEND immune score and PFS in 152 NSCLC patients treated with combined immunotherapy

Predictive models	Group	*n*	mPFS (mon)	95%CI
LIPI	Good	46	10.7	8.844-12.642
	Intermediate	70	5.9	4.293-7.469
	Poor	36	4.0	2.497-8.016
iSEND	Good	21	11.0	7.385-14.562
	Intermediate	105	7.4	5.842-9.008
	Poor	26	3.4	2.593-4.175
Total		152	6.6	5.251-8.016

**表 5:** 178例免疫治疗NSCLC患者PFS单因素与多因素*Cox*回归分析 Univariate and multivariate *Cox* regression analysis of PFS in 178 treated with immunotherapy NSCLC patients

Characteristics	Univariate analysis			Multivariate analysis	
	HR (95%CI)	*P*		HR (95%CI)	*P*
Age (< 65 yr *vs* ≥65 yr)	1.017 (0.734-1.410)	0.918			
Gender (Female *vs* Male)	1.310 (0.885-1.940)	0.177			
ECOG PS (0-1 *vs* 2)	1.907 (1.270-2.865)	0.002		1.275 (0.827-1.966)	0.272
Smoking (Yes *vs* No)	1.103 (0.804-1.514)	0.543			
Histology (Squamous *vs* Non-squamous)	1.030 (0.751-1.411)	0.855			
TNM stage (III *vs* IV)	1.330 (0.962-1.839)	0.085			
Lines of treatment (1 *vs* ≥2)	1.126 (0.803-1.579)	0.493			
Option of treatment (Monotherapy *vs* Combination therapy)	0.762 (0.496-1.170)	0.214			
DNLR (< 0 *vs* ≥0)	1.162 (0.844-1.600)	0.357			
NLR (< 5 *vs* ≥5)	2.318 (1.627-3.303)	< 0.001		1.440 (0.943-2.198)	0.091
dNLR (≤3 *vs* > 3)	1.862 (1.352-2.565)	< 0.001		1.546 (1.068-2.238)	0.021
LDH (≤245 IU/L *vs* > 245 IU/L)	2.399 (1.724-3.339)	< 0.001		2.065 (1.444-2.954)	< 0.001

**表 6:** 152例免疫联合治疗NSCLC患者PFS单因素与多因素*Cox*回归分析 Univariate and multivariate *Cox* regression analysis of PFS in 152 NSCLC patients treated with combined immunotherapy

Characteristics	Univariate analysis			Multivariate analysis	
	HR (95%CI)	*P*		HR (95%CI)	*P*
Age (< 65 yr *vs* ≥65 yr)	1.01 (0.706-1.446)	0.955			
Gender (Female *vs* Male)	1.423 (0.916-2.21)	0.116			
ECOG PS (0-1 *vs* 2)	1.827 (1.172-2.848)	0.008		1.241 (0.769-2.002)	0.376
Smoking (Yes *vs* No)	1.177 (0.835-1.661)	0.352			
Histology (Squamous *vs* Non-squamous)	1.057 (0.749-1.493)	0.751			
TNM stage (III *vs* IV)	1.256 (0.998-1.579)	0.052			
Lines of treatment (1 *vs* ≥2)	1.173 (0.816-1.684)	0.389			
DNLR (< 0 *vs* ≥0)	1.149 (0.81-1.63)	0.437			
NLR (< 5 vs ≥5)	2.405 (1.636-3.537)	< 0.001		1.408 (0.886-2.238)	0.148
dNLR (≤3 *vs* > 3)	2.061 (1.448-2.934)	< 0.001		1.779 (1.187-2.666)	0.005
LDH (≤245 IU/L *vs* > 245 IU/L)	2.179 (1.519-3.124)	< 0.001		1.926 (1.304-2.845)	< 0.001

## 3 讨论

免疫治疗已广泛用于晚期NSCLC患者的临床治疗^[5]^，但仍有部分患者无法从中获益，且免疫治疗可能伴有明显的不良反应及较重的经济负担。PD-L1表达水平作为获批指导免疫治疗的标志物，因其价格昂贵、取材困难、检测方法不同，应用于临床范围有限，并且PD-L1表达与免疫治疗的预后并不总是相关^[6, 7]^。因此寻找易获得、便捷、准确的指标用于评估治疗疗效及预后，对协助筛选能获益人群有重要意义。LIPI及iSEND免疫评分均由外国学者提出，由较易获得的性别、ECOG PS评分、NLR、DNLR、dNLR、LDH构成，但对中国晚期NSCLC患者免疫治疗的预测价值仍缺乏验证。两种预测系统中的血液相关指标均与机体炎症反应相关。全身炎症反应被认为与肿瘤的发生、进展密切相关，外周血中性粒细胞、淋巴细胞、LDH均可反映全身炎症反应程度^[8-10]^。外周血中性粒细胞可通过分泌细胞因子如白细胞介素1（interleukin 1, IL-1）、IL-6、血管内皮生长因子（vascular endothelial growth factor, VEGF）、肿瘤坏死因子α（tumor necrosis factor, TNF-α）等促进肿瘤细胞增殖、诱导肿瘤血管生成，进而促成肿瘤进展和转移，并且能够抑制免疫系统中自然杀伤细胞和活化T细胞的抗肿瘤反应^[11, 12]^。淋巴细胞是免疫监测及抗肿瘤反应的主要效应细胞，ICIs通过激活T淋巴细胞和阻断负性调节因子来增强抗肿瘤免疫反应^[13]^。LDH由快速生长的肿瘤细胞产生，可以反映实体瘤患者的肿瘤负担和炎症状态^[14, 15]^。由血细胞计数得出的NLR和dNLR已被用于衡量多种肿瘤患者的机体炎症状态^[9, 16-18]^。DNLR为不同时间点NLR差值，可反映机体炎症反应程度的变化^[19]^。

本研究对178例接受免疫治疗的晚期NSCLC患者进行iSEND免疫评分及LIPI评分，分析两种模型对疗效及预后的预测价值。与既往国外研究一致^[20, 21]^，两种模型对ORR、DCR、PFS均有较好的预测价值。但目前尚无两种模型预测价值对比相关研究，在本研究中LIPI的预测价值均优于iSEND评分系统。在全组患者的生存分析中，iSEND免疫评分分组良好、中等、较差组的PFS依次减短；同样，LIPI分组良好、中等、较差组的PFS依次减短。由于我国免疫治疗药物价格及医保政策影响，本研究中患者几乎全部为PD-1单抗治疗，且多数为联合治疗，我们对152例免疫联合治疗患者进行生存分析，发现不同iSEND免疫评分分组及LIPI分组的PFS同样具有差异。我们同时对患者进行了*Cox*回归分析，发现dNLR、LDH在全组患者及联合治疗亚组中均为PFS的独立预测因素。

既往多项研究表明LIPI、iSEND免疫评分对晚期NSCLC患者应用免疫治疗的预后具有预测价值。Mezquita等^[3]^提出LIPI后，在应用免疫治疗的NSCLC患者中验证了LIPI对疗效及预后的预测价值；Hopkins等^[22]^的研究也发现在免疫联合治疗方案的NSCLC患者中，良好LIPI组均与更好的OS、PFS相关，较差LIPI组与更短的OS、PFS相关。与既往结果一致，本研究中同样发现在全组患者中良好LIPI组与更好的PFS相关；并且在免疫联合治疗亚组中，良好LIPI组仍与更好的PFS相关。国内一项研究^[23]^表明，在应用非手术治疗的老年NSCLC患者中LIPI分组同样与患者预后相关，虽然未单独在免疫治疗中进行分析，但整体结果仍可显示LIPI在NSCLC患者中的预测价值。Park等^[20]^研究发现iSEND免疫评分系统对应用免疫治疗的NSCLC患者的OS及PFS均具有预测价值，评分较差组的OS及PFS明显较中等组及良好组短。与该研究结果一致，在本研究中应用免疫联合治疗的患者iSEND免疫评分不同的三组患者的PFS具有明显差异。Kazandjian等^[24]^、Park等^[20]^的研究分别发现在应用免疫单药治疗的NSCLC患者中，良好LIPI组、良好iSEND评分组与更好的PFS相关，但本研究中应用免疫单药患者LIPI、iSEND免疫评分均与PFS无明显相关，与既往研究结果有所不同，考虑可能与本研究中应用免疫单药治疗病例过少、存在误差有关，这两种预测模型在我国晚期NSCLC患者应用免疫单药治疗中的预测价值需进一步验证。

本研究发现LIPI在疗效及预后的预测效果均高于iSEND免疫评分。两种模型均有简单、易获得、廉价等优点，但iSEND免疫评分系统所纳入的性别、DNLR在本研究中未表现出与PFS的相关性；相反，我们通过多因素分析发现dNLR、LDH为PFS的独立预测因素，二者均为LIPI的构成指标，这可能与LIPI预测效果更好相关。但在王桂春等^[25]^的研究中，免疫联合治疗患者中iSNED免疫评分良好组与较好的生存独立相关，与本研究结果有所不同，可能与该研究在进行评分时因ECOG PS≥2分的患者较少，更改为ECOG PS≥1分记为1分，使评分优组患者一般情况更好，并且将评分中等与较差合为一组进行分析相关。本研究仍有一些不足之处，作为单中心回顾性研究，存在偏倚可能；随访时间较短，暂未能收集到完整OS数据。

综上所述，通过对真实世界的数据进行分析，我们发现LIPI及iSEND免疫评分在晚期应用免疫治疗NSCLC患者中，对疗效及预后均具有较好的预测价值；其中LIPI的预测价值优于iSEND免疫评分。LIPI由临床常见指标组成，临床可考虑结合LIPI评分为患者制定个性化治疗方案。未来可通过多中心前瞻性研究进一步研究LIPI及iSEND免疫评分在我国晚期NSCLC免疫治疗患者中的应用价值。
